# Supplementary figures and images for: Beneficial endophytic fungi improve the yield and quality of Salvia miltiorrhiza by performing different ecological functions
Source: PeerJ. 2024 Feb 22;12:e16959. doi: 10.7717/peerj.16959 (PMC10894594; doi:10.7717/peerj.16959)

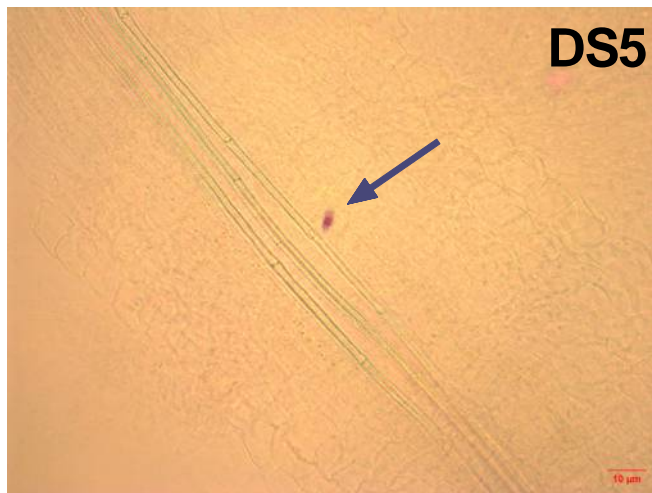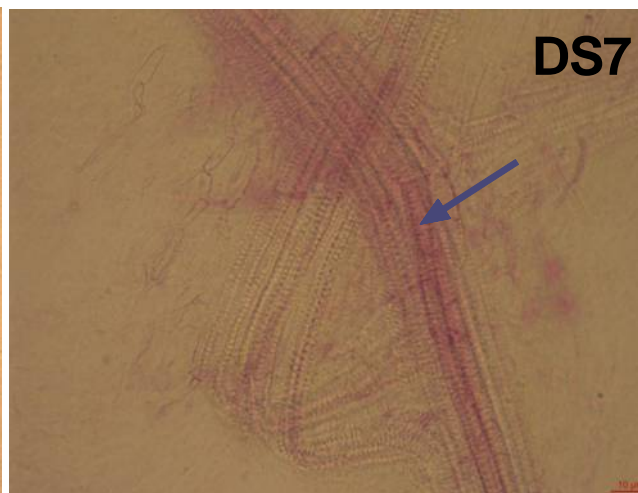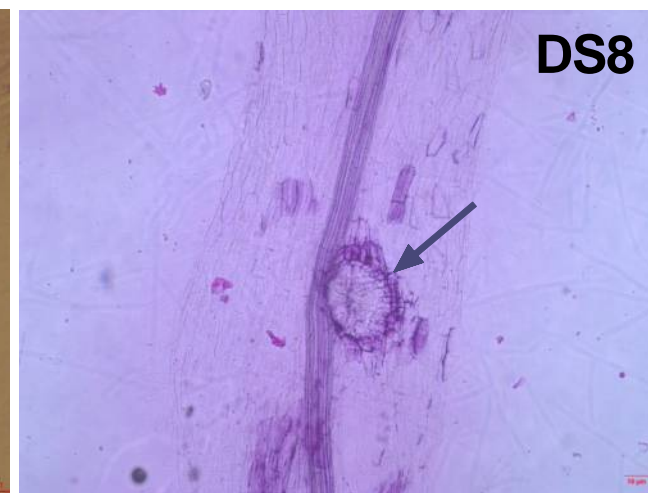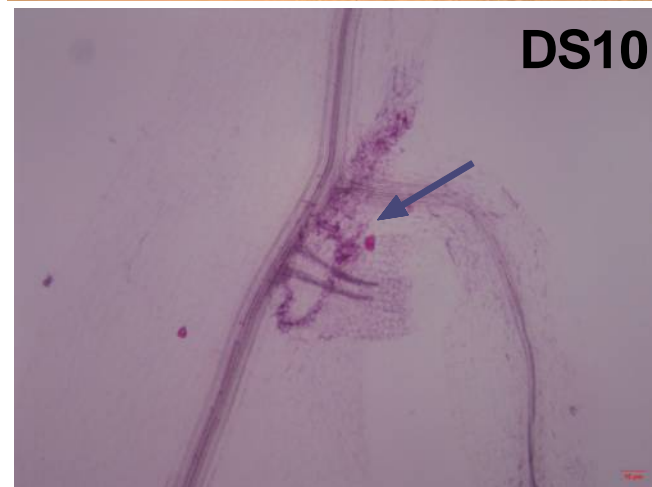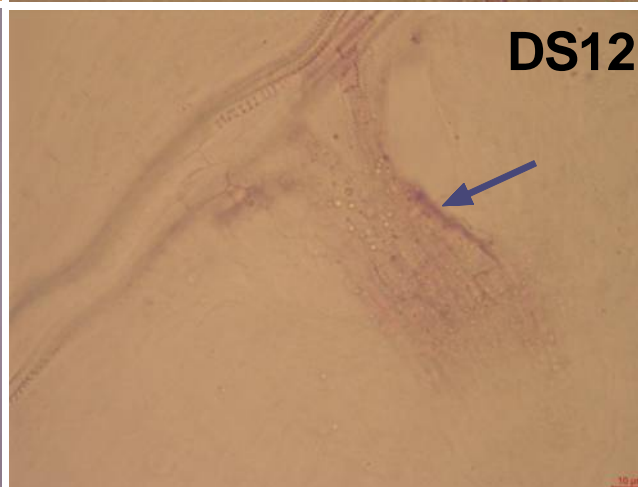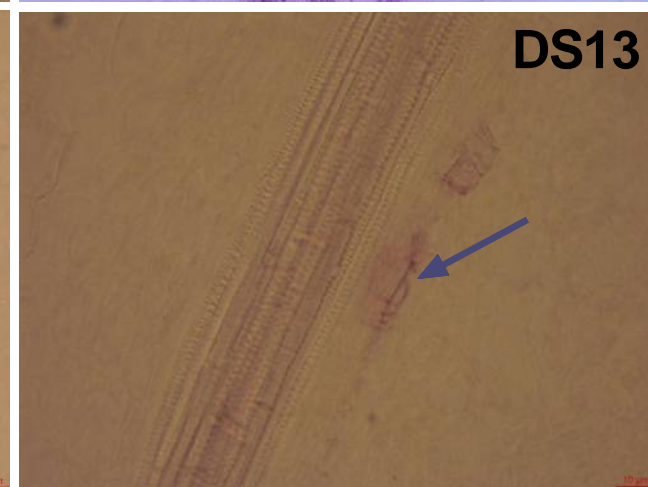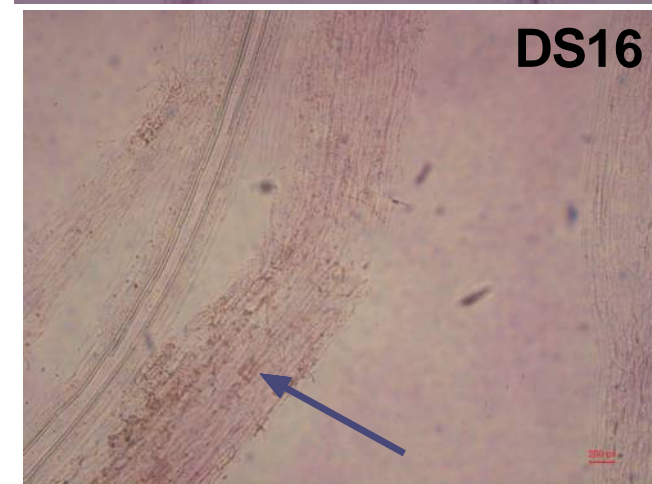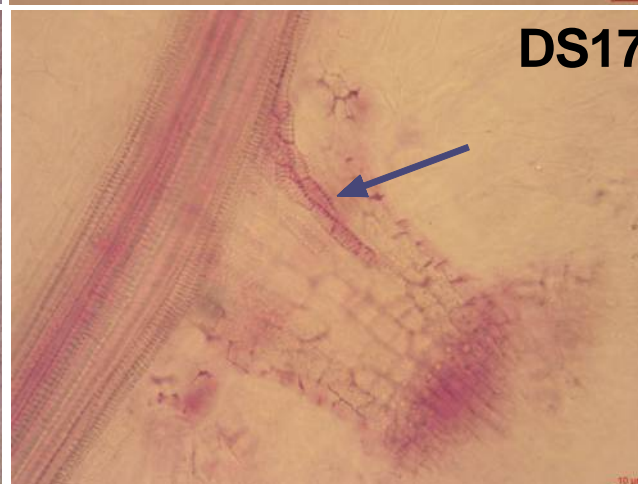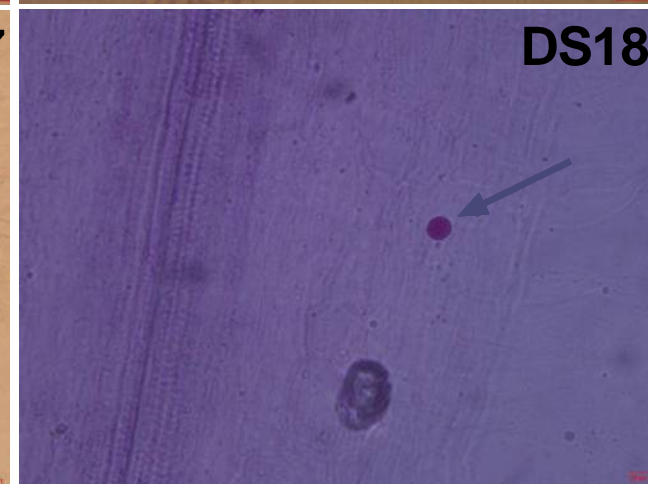

Supplement: Figure S1 [file peerj-12-16959-s001.pdf]

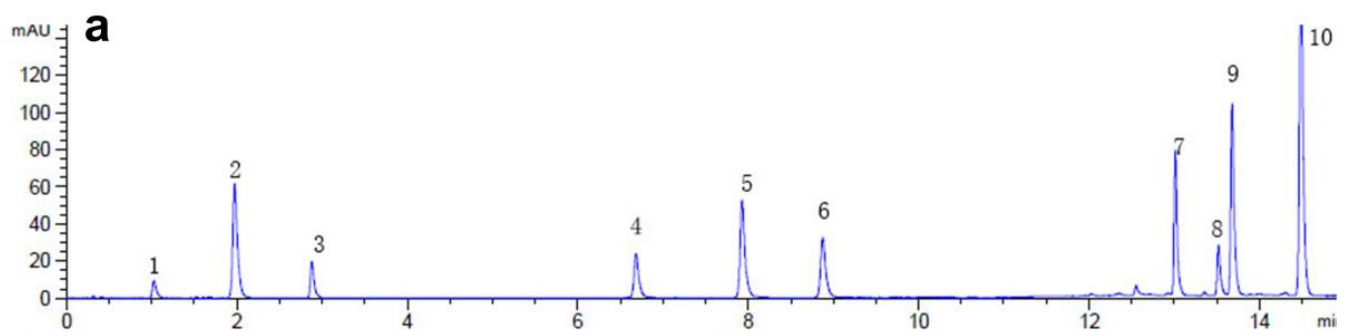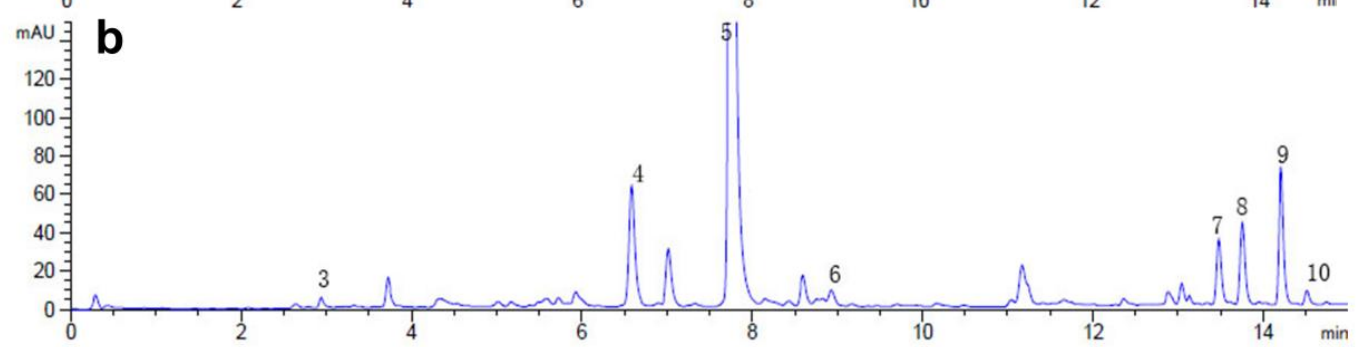

Supplement: Figure S2 — 3: Caffeic acid; 4: Rosmarinic acid; 5: Salvianolic acid B; 6: Salvianolic acid A; 7: Dihydrotanshinone; 8: Tanshinone I; 9: Cryptotanshinone; 10: Tanshinone IIA. [file peerj-12-16959-s002.pdf]
