# Supplementary material for: Beneficial endophytic fungi improve the yield and quality of Salvia miltiorrhiza by performing different ecological functions
Source: PeerJ. 2024 Feb 22;12:e16959. doi: 10.7717/peerj.16959 (PMC10894594; doi:10.7717/peerj.16959)
Supplement: Table S1 [file peerj-12-16959-s003.docx]

Table S1-1. UPLC system precision test results

| **sample number** | **Active ingredients peak area** | | | | | | | |
| --- | --- | --- | --- | --- | --- | --- | --- | --- |
|  | **Caffeic acid** | **Rosmarinic acid** | **Salvianolic acid B** | **Salvianolic acid A** | **Dihydrotanshinone** | **Tanshinone I** | **Cryptotanshinone** | **Tanshinone IIA** |
| 1 | 101.96 | 331.12 | 275.81 | 207.63 | 990.39 | 11.49 | 186.85 | 576.20 |
| 2 | 99.64 | 324.21 | 273.61 | 204.72 | 973.40 | 12.98 | 183.25 | 567.08 |
| 3 | 100.48 | 327.09 | 276.45 | 206.63 | 981.20 | 12.90 | 185.06 | 571.42 |
| 4 | 99.77 | 323.75 | 273.23 | 202.69 | 972.43 | 12.13 | 183.74 | 567.51 |
| 5 | 99.40 | 324.49 | 274.62 | 204.94 | 973.93 | 11.94 | 184.10 | 568.03 |
| 6 | 100.09 | 325.39 | 273.48 | 204.13 | 974.60 | 13.36 | 183.53 | 568.90 |
| ‾x | 100.22 | 326.01 | 274.53 | 205.12 | 977.66 | 12.47 | 184.42 | 569.86 |
| RSD（%） | 0.90 | 0.80 | 0.50 | 0.90 | 0.70 | 5.80 | 0.70 | 0.60 |

Table S1-2. UPLC repeatability test results

| **sample number** | **Active ingredients content（mg·g^-1^）** | | | | | | | |
| --- | --- | --- | --- | --- | --- | --- | --- | --- |
|  | **Caffeic acid** | **Rosmarinic acid** | **Salvianolic acid B** | **Salvianolic acid A** | **Dihydrotanshinone** | **Tanshinone I** | **Cryptotanshinone** | **Tanshinone IIA** |
| 1 | 0.099 | 0.664 | 36.995 | 0.117 | 0.068 | 0.124 | 0.143 | 0.085 |
| 2 | 0.099 | 0.641 | 38.857 | 0.112 | 0.068 | 0.135 | 0.148 | 0.083 |
| 3 | 0.099 | 0.666 | 36.515 | 0.120 | 0.068 | 0.135 | 0.160 | 0.086 |
| 4 | 0.098 | 0.669 | 37.638 | 0.114 | 0.076 | 0.140 | 0.154 | 0.085 |
| 5 | 0.099 | 0.633 | 38.826 | 0.114 | 0.068 | 0.135 | 0.152 | 0.084 |
| 6 | 0.098 | 0.649 | 35.869 | 0.114 | 0.068 | 0.134 | 0.148 | 0.085 |
| ‾x | 0.099 | 0.654 | 37.450 | 0.115 | 0.070 | 0.134 | 0.151 | 0.085 |
| RSD（%） | 0.4 | 2.3 | 3.3 | 2.5 | 4.6 | 3.9 | 3.8 | 1.4 |

Table S1-3. UPLC Stability test results

| **Measuring time** | **Active ingredients content（mg·g^-1^）** | | | | | | | |
| --- | --- | --- | --- | --- | --- | --- | --- | --- |
|  | **Caffeic acid** | **Rosmarinic acid** | **Salvianolic acid B** | **Salvianolic acid A** | **Dihydrotanshinone** | **Tanshinone I** | **Cryptotanshinone** | **Tanshinone IIA** |
| 18-3(0h) | 0.115 | 0.671 | 35.929 | 0.116 | 0.069 | 0.129 | 0.150 | 0.081 |
| 18-3(4h) | 0.113 | 0.659 | 36.054 | 0.115 | 0.067 | 0.149 | 0.159 | 0.090 |
| 18-3(8h) | 0.111 | 0.665 | 36.463 | 0.119 | 0.069 | 0.135 | 0.152 | 0.085 |
| 18-3(12h) | 0.109 | 0.660 | 36.448 | 0.119 | 0.076 | 0.133 | 0.152 | 0.085 |
| 18-3(24h) | 0.109 | 0.665 | 36.445 | 0.120 | 0.076 | 0.135 | 0.152 | 0.085 |
| ‾x | 0.111 | 0.664 | 36.268 | 0.118 | 0.072 | 0.136 | 0.153 | 0.085 |
| RSD（%） | 2.2 | 0.72 | 0.71 | 1.84 | 6.21 | 5.41 | 2.15 | 3.59 |

Table S1-4. UPLC Stability test results

| **Active ingredients** | **sample number** | **Sample weight（g）** | **original value（mg）** | **quantity added（mg）** | **measured quantity（mg）** | **rate of recovery（%）** | **RSD（%）** |
| --- | --- | --- | --- | --- | --- | --- | --- |
| **Caffeic acid** | 1 | 0.5020 | 0.0126 | 0.0174 | 0.0299 | 99.5200 | 0.71 |
|  | 2 | 0.5100 | 0.0127 | 0.0174 | 0.0301 | 100.1100 |  |
|  | 3 | 0.5030 | 0.0129 | 0.0174 | 0.0305 | 101.1000 |  |
|  | 4 | 0.5040 | 0.0125 | 0.0174 | 0.0299 | 100.0100 |  |
|  | 5 | 0.5070 | 0.0121 | 0.0174 | 0.0297 | 101.1200 |  |
| **Rosmarinic acid** | 1 | 0.5020 | 0.1760 | 0.1011 | 0.2793 | 102.2200 | 1.06 |
|  | 2 | 0.5100 | 0.1761 | 0.1011 | 0.2791 | 101.8300 |  |
|  | 3 | 0.5030 | 0.1759 | 0.1011 | 0.2792 | 102.1300 |  |
|  | 4 | 0.5040 | 0.1765 | 0.1011 | 0.2819 | 104.2900 |  |
|  | 5 | 0.5070 | 0.1770 | 0.1011 | 0.2818 | 103.6900 |  |
| **Salvianolic acid B** | 1 | 0.5020 | 5.4033 | 2.6284 | 2.4820 | 94.4300 | 2.50 |
|  | 2 | 0.5100 | 5.4038 | 2.6284 | 7.9760 | 97.8600 |  |
|  | 3 | 0.5030 | 5.4032 | 2.6284 | 7.9538 | 97.0400 |  |
|  | 4 | 0.5040 | 5.4030 | 2.6284 | 8.0600 | 101.0900 |  |
|  | 5 | 0.5070 | 5.4039 | 2.6284 | 8.0031 | 98.8900 |  |
| **Salvianolic acid A** | 1 | 0.5020 | 0.0255 | 0.0420 | 0.0680 | 101.2100 | 0.83% |
|  | 2 | 0.5100 | 0.0253 | 0.0420 | 0.0675 | 100.5800 |  |
|  | 3 | 0.5030 | 0.0262 | 0.0420 | 0.0688 | 101.4200 |  |
|  | 4 | 0.5040 | 0.0260 | 0.0420 | 0.0690 | 102.2700 |  |
|  | 5 | 0.5070 | 0.0257 | 0.0420 | 0.0677 | 100.0700 |  |
| **Dihydrotanshinone** | 1 | 0.5020 | 0.0210 | 0.0202 | 0.0389 | 88.6800 | 0.24 |
|  | 2 | 0.5100 | 0.0200 | 0.0202 | 0.0380 | 88.8900 |  |
|  | 3 | 0.5030 | 0.0203 | 0.0202 | 0.0382 | 88.7900 |  |
|  | 4 | 0.5040 | 0.0206 | 0.0202 | 0.0386 | 89.0500 |  |
|  | 5 | 0.5070 | 0.0201 | 0.0202 | 0.0381 | 89.2200 |  |
| **Tanshinone I** | 1 | 0.5020 | 0.1206 | 0.0076 | 0.1281 | 98.7700 | 4.70 |
|  | 2 | 0.5100 | 0.1205 | 0.0076 | 0.1275 | 92.4400 |  |
|  | 3 | 0.5030 | 0.1202 | 0.0076 | 0.1270 | 89.3900 |  |
|  | 4 | 0.5040 | 0.1203 | 0.0076 | 0.1271 | 89.2100 |  |
|  | 5 | 0.5070 | 0.1207 | 0.0076 | 0.1274 | 88.2400 |  |
| **Cryptotanshinone** | 1 | 0.5020 | 0.0780 | 0.0649 | 0.1342 | 86.6100 | 2.83 |
|  | 2 | 0.5100 | 0.0757 | 0.0649 | 0.1350 | 91.4100 |  |
|  | 3 | 0.5030 | 0.0769 | 0.0649 | 0.1375 | 93.3700 |  |
|  | 4 | 0.5040 | 0.0776 | 0.0649 | 0.1369 | 91.4400 |  |
|  | 5 | 0.5070 | 0.0757 | 0.0649 | 0.1355 | 92.1200 |  |
| **Tanshinone IIA** | 1 | 0.5020 | 0.0667 | 0.0753 | 0.1373 | 93.7500 | 1.93 |
|  | 2 | 0.5100 | 0.0676 | 0.0753 | 0.1401 | 96.3100 |  |
|  | 3 | 0.5030 | 0.0685 | 0.0753 | 0.1424 | 98.1100 |  |
|  | 4 | 0.5040 | 0.0690 | 0.0753 | 0.1427 | 97.8400 |  |
|  | 5 | 0.5070 | 0.0668 | 0.0753 | 0.1407 | 98.0900 |  |
