# Supplementary material for: Beneficial endophytic fungi improve the yield and quality of Salvia miltiorrhiza by performing different ecological functions
Source: PeerJ. 2024 Feb 22;12:e16959. doi: 10.7717/peerj.16959 (PMC10894594; doi:10.7717/peerj.16959)
Supplement: Supplemental Information 5 [file peerj-12-16959-s005.docx]

>DS5

CTCTGGGTCCACCTCCCACCCGTGTTTATCGTACCTTGTTGCTTCGGCGGGCCCGCCGTCATGGCCGCCGGGGGGCACTC

GCCCCCGGGCCCGCGCCCGCCGAAGACACCATTGAACTCTGTCTGAAGATTGCAGTCTGAGCGATTAGCTAAATCAGTTA

AAACTTTCAACAACGGATCTCTTGGTTCCGGCATCGATGAAGAACGCAGCGAAATGCGATAAGTAATGTGAATTGCAGAA

TTCAGTGAATCATCGAGTCTTTGAACGCACATTGCGCCCCCTGGTATTCCGGGGGGCATGCCTGTCCGAGCGTCATTGCT

GCCCTCAAGCACGGCTTGTGTGTTGGGCCCCCGCCCCCCGGTTCCGGGGGGCGGGCCCGAAAGGCAGCGGCGGCACCGCG

TCCGGTCCTCGAGCGTATGGGGCTTCGTCACCCGCTCTGTAGGCCCGGCCGGCGCCCGCCGGCGACCCCAATCAATCTTT

CCAGGTTGACCTCGGATCAGGTAGGGATACCCGCTGAACTTAAGCATATCAATAAAGCGGAGGAAA

>DS7

CGGAGTTAAGGTCTCGTTGGTGACCAGCGGAGGGATCATTACCGAGTGTAAAAACTCCCAAACCATTGTGAACCTACCAC

TGTTGCTTCGGCGGCCTCGCCCCGGGCGCGTTCGCGCGGCCCGGACCCAGGCGTCCGCCGGAGGCTCCAAACTCTTGTCT

TTTAGTGTATTTCTGAGTGGCATAAGCAAATAAATCAAAACTTTCAGCAACGGATCTCTTGGTTCTGGCATCGATGAAGA

ACGCAGCAAAATGCGATAAGTAATGTGAATTGCAGAATTCAGTGAATCATCGAATCTTTGAACGCACATTGCGCCCGCCA

GTATTCTGGCGGGCATGCCTGTCTGAGCGTCATTTCAACCCTCAGGACCCGTTCGCGGGACCTGGCGTTGGGGATCAGCC

TGCCCCTGGCGGCGGCTGGCCCTGAAATCCAGTGGCGGTTCCCTCGCGAACTCCTCCGTGCAGTAATTAAACCTCTCGCG

GCAGGATAGCGGTTGAACCACGCCGTTAAACCCCCCACTTCTCAAGGTTGACCTCAGATCAGGTAGGAATACCCGCTGAA

CTTAAGCATATCAA

>DS8

TTACGAGGTGAGGGCTCTGGGTCACCTCCCACCCGTGTTTATCGTACCTTGTTGCTTCGGCGGGCCCGCCGTCATGGCCG

CCGGGGGGCACTCGCCCCCGGGCCCGCGCCCGCCGAAGACACCATTGAACTCTGTCTGAAGATTGCAGTCTGAGCGATTA

GCTAAATCAGTTAAAACTTTCAACAACGGATCTCTTGGTTCCGGCATCGATGAAGAACGCAGCGAAATGCGATAAGTAAT

GTGAATTGCAGAATTCAGTGAATCATCGAGTCTTTGAACGCACATTGCGCCCCCTGGTATTCCGGGGGGCATGCCTGTCC

GAGCGTCATTGCTGCCCTCAAGCACGGCTTGTGTGTTGGGCCCCCGCCCCCCGGTTCCGGGGGGCGGGCCCGAAAGGCAG

CGGCGGCACCGCGTCCGGTCCTCGAGCGTATGGGGCTTCGTCACCCGCTCTGTAGGCCCGGCCGGCGCCCGCCGGCGACC

CCAATCAATCTTTCCAGGTTGACCTCGGATCAGGTAGGGATACCCGCTGAACTTAAGCATATCAAAAAA

>DS10

CCGGAGAAAAAAAAACGCGAGGATGACCTGCGGAGGGAAATTATCGAGTTTTTAACTCTTAAACCATATGTGAACGTACC

TTTTCTAGCTGCTTTGGCAGGTGCCTCTCGGGGCTTCTGCCGGTAGCATTTATAAACTCTTTATATTTCTATAGAATTAT

TCATTGCTGAGTGGCATTAACTAAATAAGTTAAAACTTTCAACAACGGATCTCTTGGCTCTAGCATCGATGAAGAACGCA

GCGAAATGCGATAAGTAATGTGAATTGCAGAATTCAGTGAATCATCGAATCTTTGAACGCACATTGCGCCTGGCAGTATT

CTGCCAGGCATGCCTGTCCGAGCGTCATTTCACCACTCAAGCTCTGCTTGGTGTTGGAGGACCCGCGTTTAGTCGCGGGC

CGCCGAAATGCATCGGCTGTTGTATATACAGCTTCCCTGTGTAGTAAATGCTTAGCTTTACACTTTGAAACTTTTATATA

ACATGCCGAAAAACCCTTAACTTTTGAAAGGTTGACCTCGGATCAGGTAGGAATACCCGCTGAACTTAAGCATATCAATA

AGCGGAGGA

>DS12

TAACGAGTGTAAACTCCCAACATTGTGAACCTACCACTGTTGCTTCGGCGGCCTCGCCCCGGGCGCGTTCGCGCGGCCCG

GACCCAGGCGTCCGCCGGAGGCTCCAAACTCTTGTCTTTTAGTGTATTTCTGAGTGGCATAAGCAAATAAATCAAAACTT

TCAGCAACGGATCTCTTGGTTCTGGCATCGATGAAGAACGCAGCAAAATGCGATAAGTAATGTGAATTGCAGAATTCAGT

GAATCATCGAATCTTTGAACGCACATTGCGCCCGCCAGTATTCTGGCGGGCATGCCTGTCTGAGCGTCATTTCAACCCTC

AGGACCCGTTCGCGGGACCTGGCGTTGGGGATCAGCCTGCCCCTGGCGGCGGCTGGCCCTGAAATCCAGTGGCGGTTCCC

TCGCGAACTCCTCCGTGCAGTAATTAAACCTCTCGCGGCAGGATAGCGGTTGAACCACGCCGTTAAACCCCCCACTTCTC

AAGGTTGACCTCAGATCAGGTAGGAATACCCGCTGAACTTAAGCATATCAAA

>DS13

AATCGACTTACACTCTCACCCTGTGACATACCTAAAACGTTGCTTCGGCGGGAACAGACGGCCCTGTAACAACGGGCCGC

CCCCGCCAGAGGACCCCTAACTCTGTTTTTATAATGTTTTTCTGAGTAAACAAGCAAATAAATTAAAACTTTCAACAACG

GATCTCTTGGCTCTGGCATCGATGAAGAACGCAGCGAAATGCGATAAGTAATGTGAATTGCAGAATTCAGTGAATCATCG

AATCTTTGAACGCACATTGCGCCCGCCAGTATTCTGGCGGGCATGCCTGTTCGAGCGTCATTACAACCCTCAGGCCCCCG

GGCCTGGCGTTGGGGATCGGCGGAAGCCCCCTGTGGGCACACGCCGTCCCTCAAATACAGTGGCGGTCCCGCCGCAGCTT

CCATTGCGTAGTAGCTAACACCTCGCAACTGGAGAGCGGCGCGGCCATGCCGTAAAACACCCAACTTCTGAATGTTGACC

TCGAATCAGGTAGGAATACCCGCTGAACTTAAGCATATCAAA

>DS16

AAATCGAGGTCACTCTCACCCTGTGAACATACCTAAAACGTTGCTTCGGCGGGAACAGACGGCCCTGTAACAACGGGCCG

CCCCCGCCAGAGGACCCCTAACTCTGTTTTTATAATGTTTTTCTGAGTAAACAAGCAAATAAATTAAAACTTTCAACAAC

GGATCTCTTGGCTCTGGCATCGATGAAGAACGCAGCGAAATGCGATAAGTAATGTGAATTGCAGAATTCAGTGAATCATC

GAATCTTTGAACGCACATTGCGCCCGCCAGTATTCTGGCGGGCATGCCTGTTCGAGCGTCATTACAACCCTCAGGCCCCC

GGGCCTGGCGTTGGGGATCGGCGGAAGCCCCCTGTGGGCACACGCCGTCCCTCAAATACAGTGGCGGTCCCGCCGCAGCT

TCCATTGCGTAGTAGCTAACACCTCGCAACTGGAGAGCGGCGCGGCCATGCCGTAAAACACCCAACTTCTGAATGTTGAC

CTCGAATCAGGTAGGAATACCCGCTGAACTTAAGCATATCAAAA

>DS17

ACTCCCAACCCACTGTGAACCTTACCTCAGTTGCCTCGGCGGGAACGCCCCGGCCGCCTGCCCCCGCGCCGGCGCCGGAC

CCAGGCGCCCGCCGCAGGGACCCCAAACTCTCTTGCATTACGCCCAGCGGGCGGAATTTCTTCTCTGAGTTGCACAAGCA

AAAACAAATGAATCAAAACTTTCAACAACGGATCTCTTGGTTCTGGCATCGATGAAGAACGCAGCGAAATGCGATAAGTA

ATGTGAATTGCAGAATTCAGTGAATCATCGAATCTTTGAACGCACATTGCGCCCGCCAGCATTCTGGCGGGCATGCCTGT

TCGAGCGTCATTTCAACCCTCGAGCCCCCCCCGGGGGCCTCGGTGTTGGGGGACGGCACACCAGCCGCCCCCGAAATGCA

GTGGCGACCCCGCCGCAGCCTCCCCTGCGTAGTAGCACACACCTCGCACCGGAGCGCGGAGGCGGTCACGCCGTAAAACG

CCCAACTTTCTTAGAGTTGACCTCGGATCAGGTAGGAATACCCGCTGAACTTAAGCATATCATAAAAAGGGGAAGGAAAG

>DS18

CTCGACTAACTCCCAACCCATGTGACATACCTACTGTTGCTTCGGCGGGATTGCCCCGGGCGCCTCGTGTGCCCCGGATC

AGGCGCCCGCCTAGGAAACTTAACTCTTGTTTTATTTTGGAATCTTCTGAGTAGTTTTTACAAATAAATAAAAACTTTCA

ACAACGGATCTCTTGGTTCTGGCATCGATGAAGAACGCAGCGAAATGCGATAAGTAATGAGAATTGCAAAATTCAGTGAA

TCATCGAATCTTTGAACGCACATTGCGCCCGCCAGTATTCTGGCGGGCATGCCTGTCTGAGCGCCATTTCAACCCTCATG

CCCCTAGGGCGTGGTGTTGGGGATCGGCCAAAGCCCGCGAGGGACGGCCGGCCCCTAAATCTAATGGCGGACCCGTCGTG

GCCTCCTCTGCGAAGTAGTGATATTCCGCATCGGAGAGCGATGAGCCCCTGCCGTTAAACCCCCAACTTTCTAAGGTTGA

CCTCAGATCAGGTAAGAATACCCGGCTGAACTTAAGCATATCAAAA
