# Supplementary material for: Beneficial endophytic fungi improve the yield and quality of Salvia miltiorrhiza by performing different ecological functions
Source: PeerJ. 2024 Feb 22;12:e16959. doi: 10.7717/peerj.16959 (PMC10894594; doi:10.7717/peerj.16959)

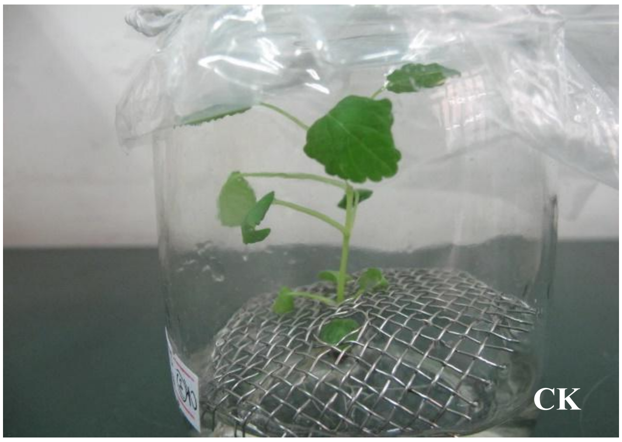

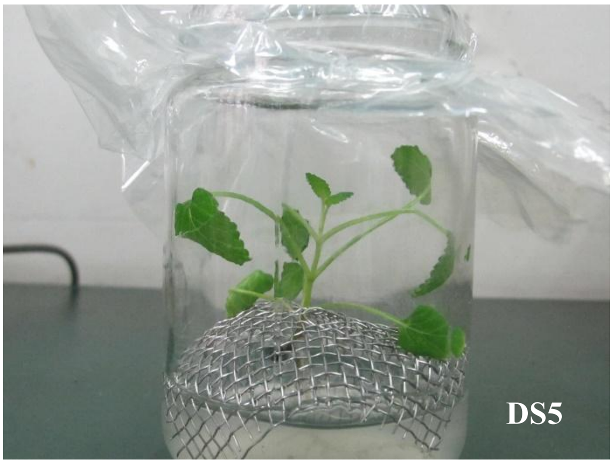

Sterile bottle cultivation:

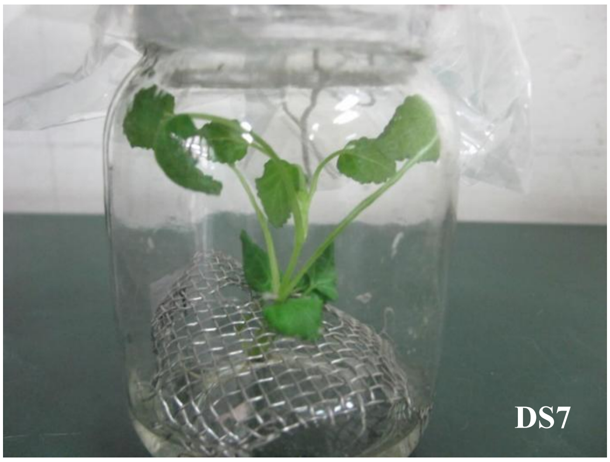

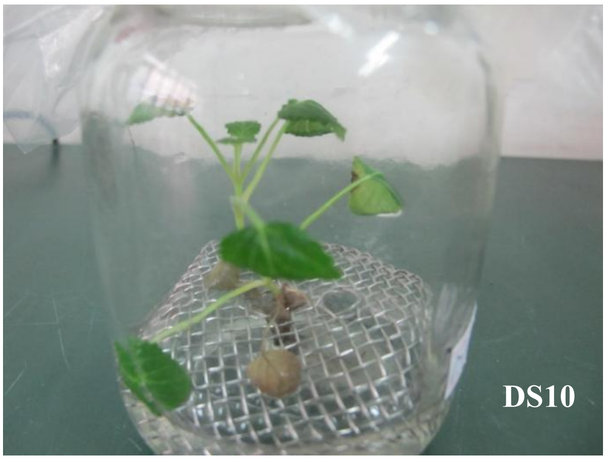

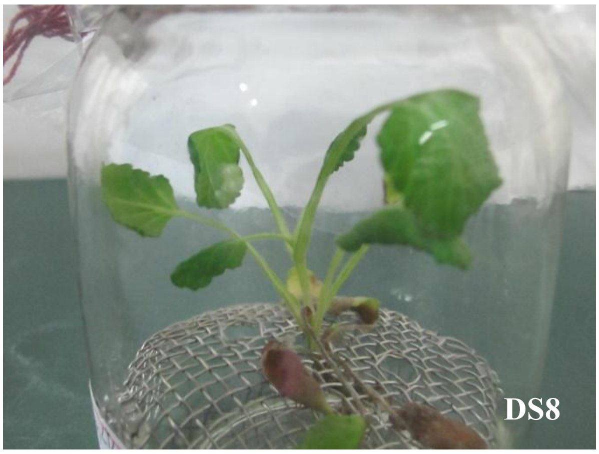

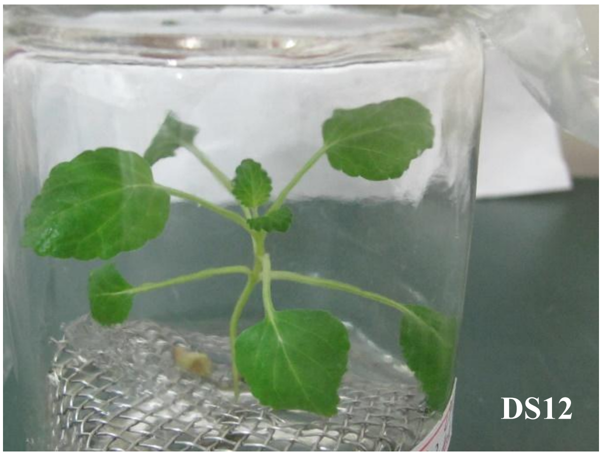

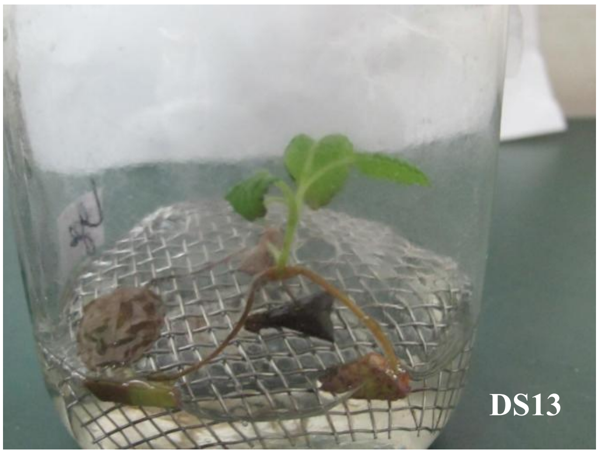

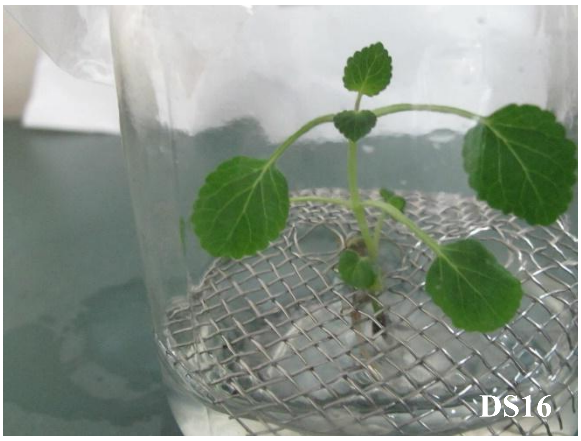

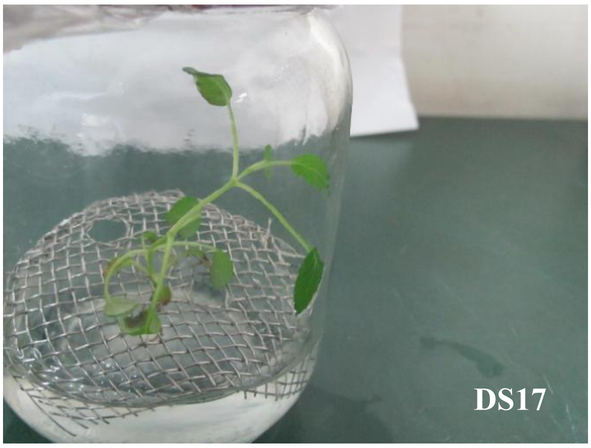

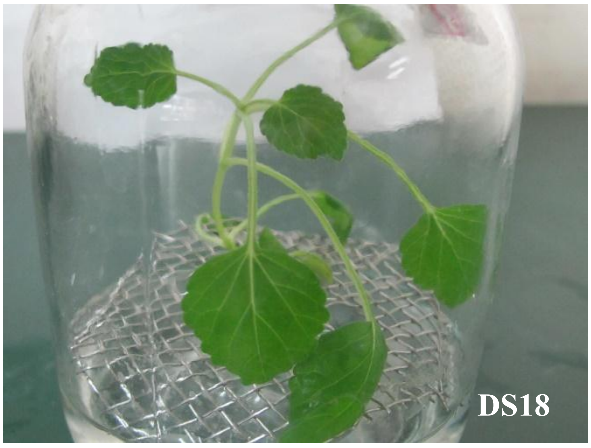

Greenhouse cultivation:

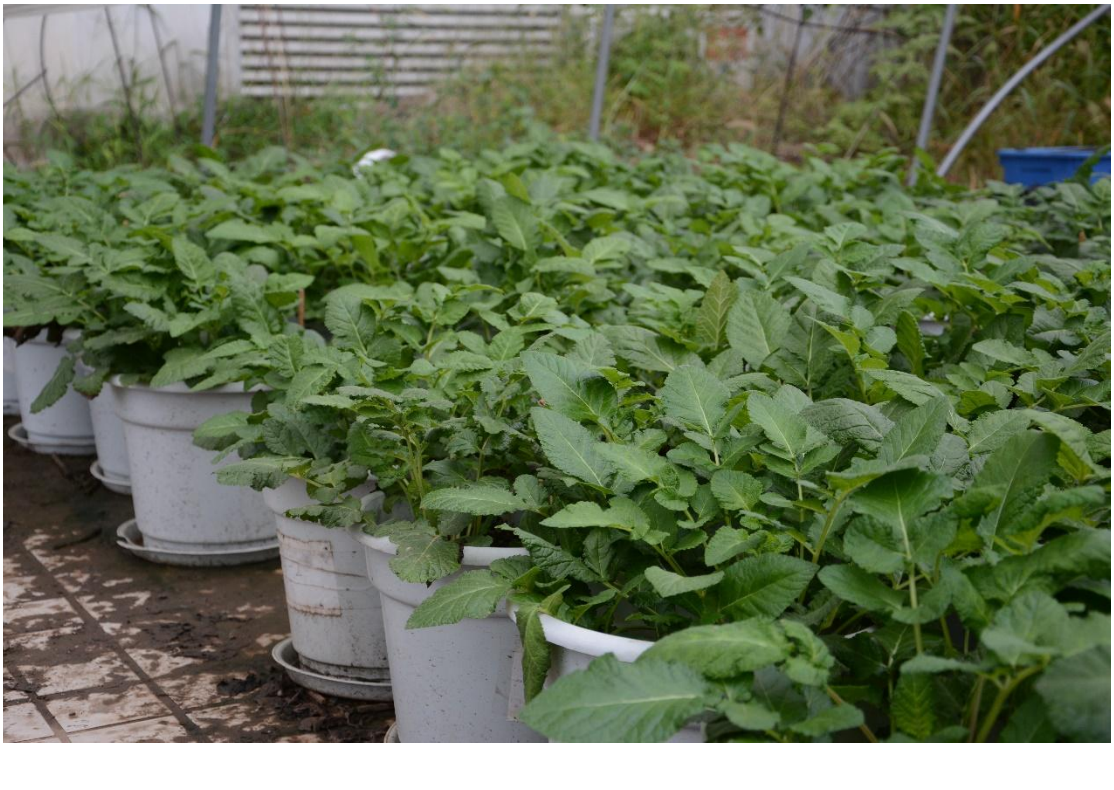

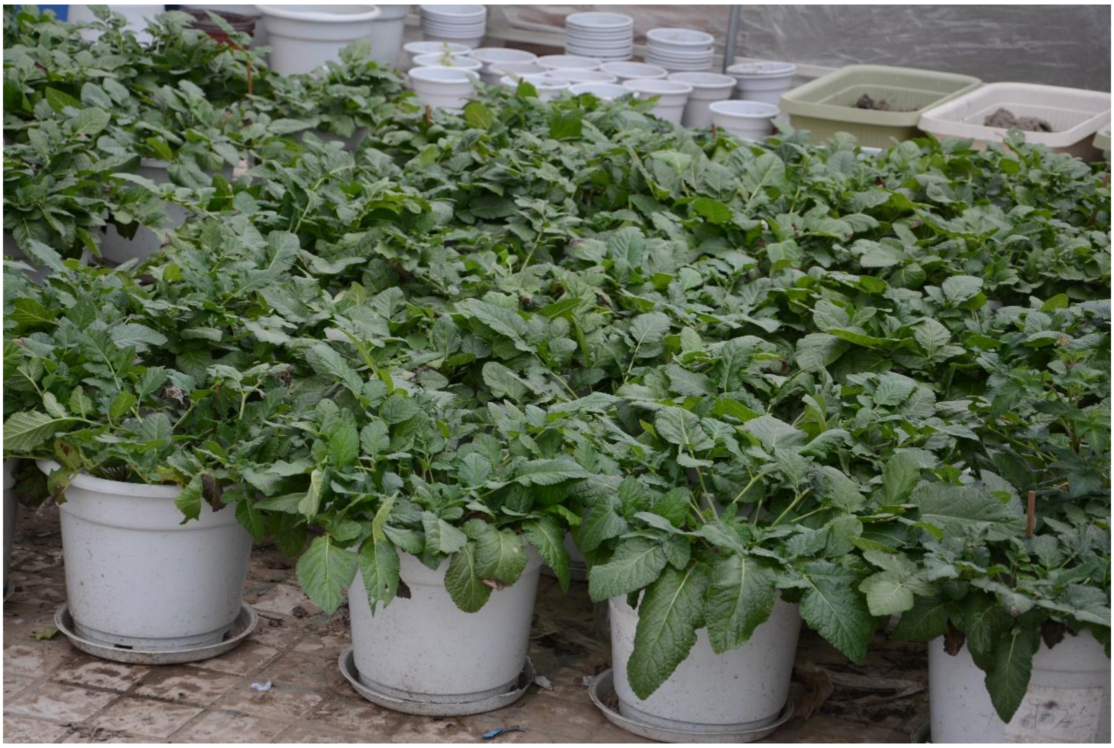

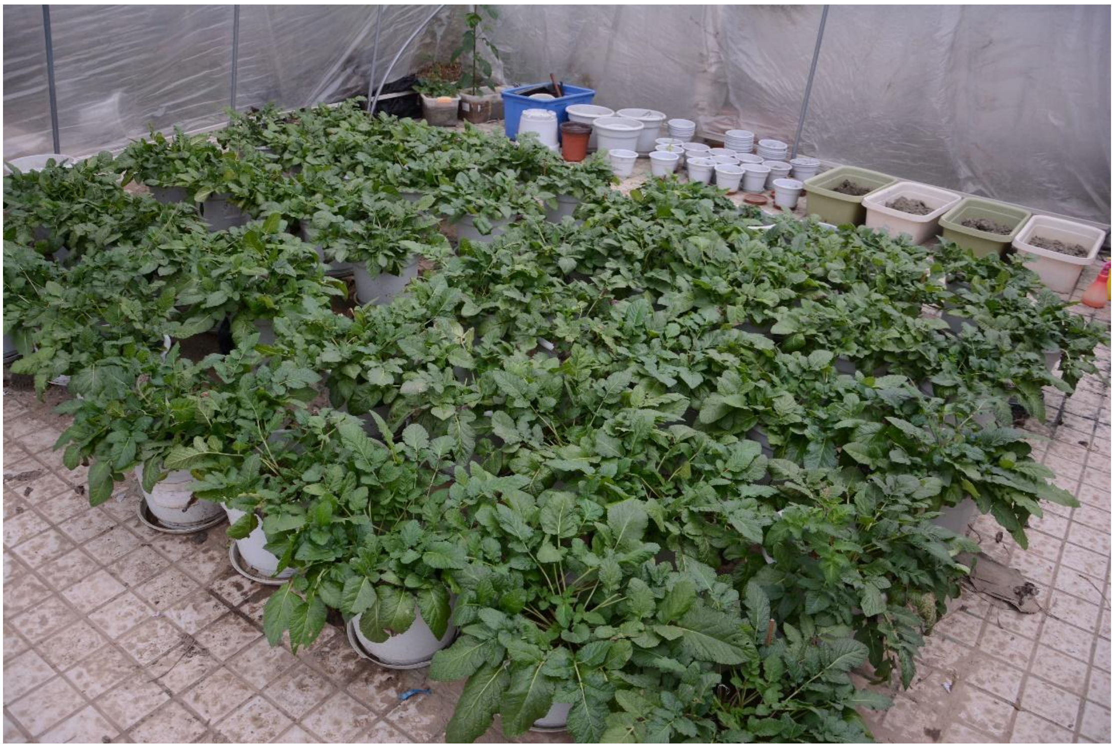

The underground part of *S. miltiorrhiza* after co-cultivation with endophytic fungi for 36 weeks in the pot.

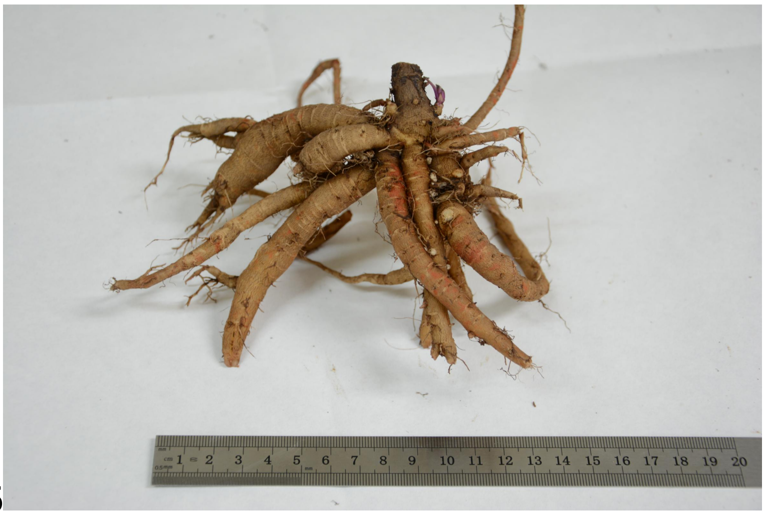

DS5

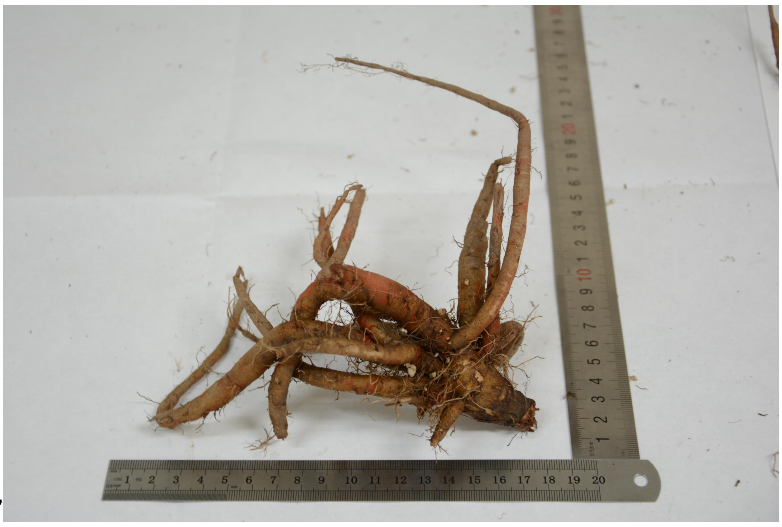

DS7

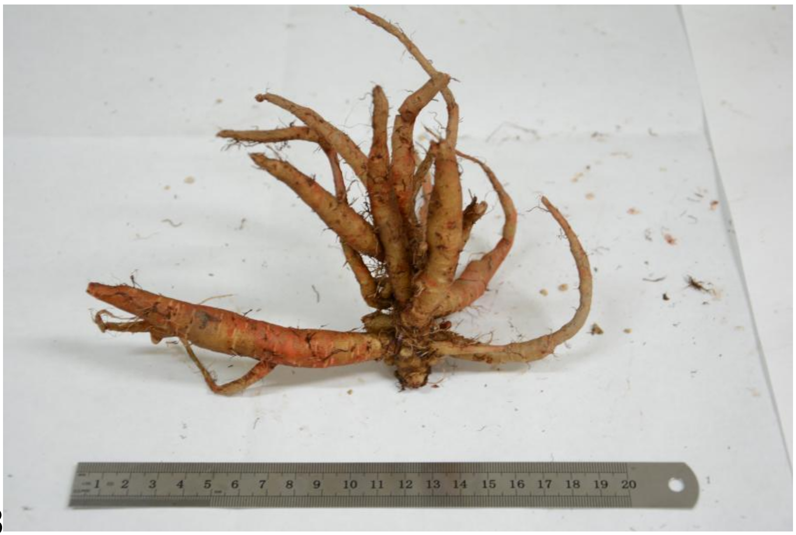

DS8

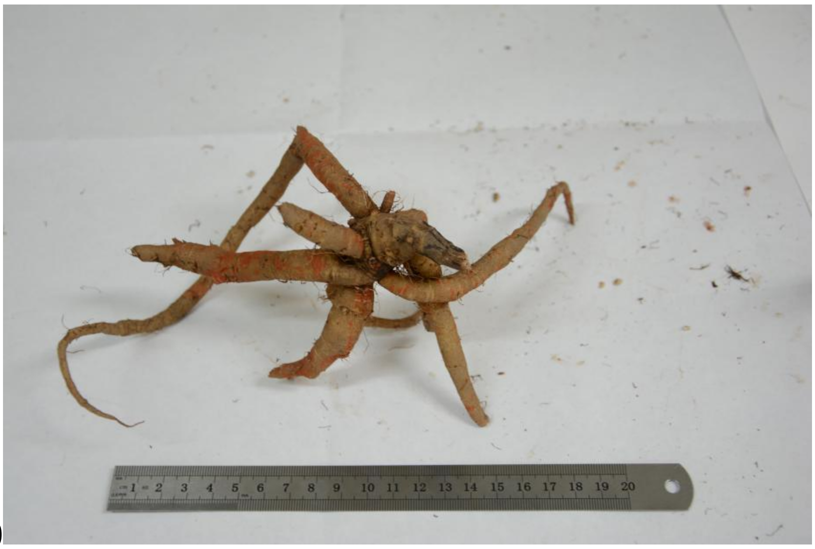
DS10

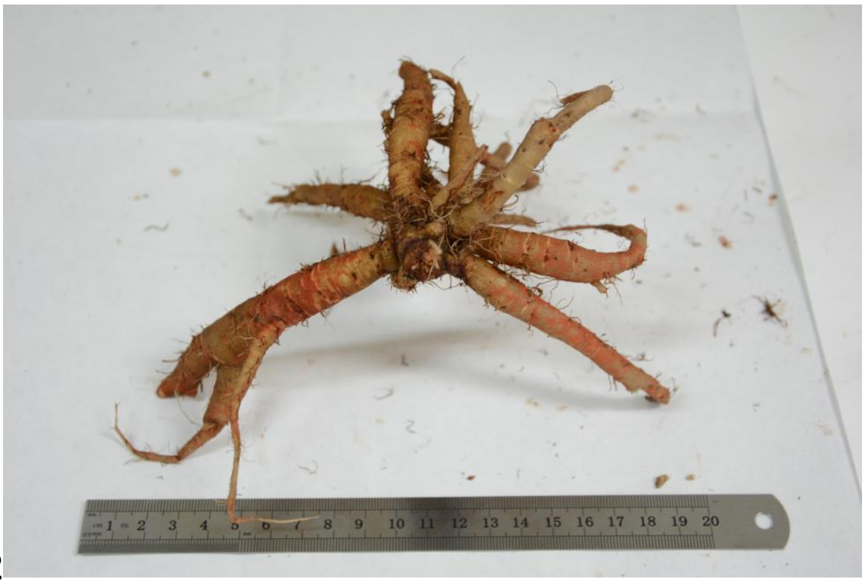
DS12

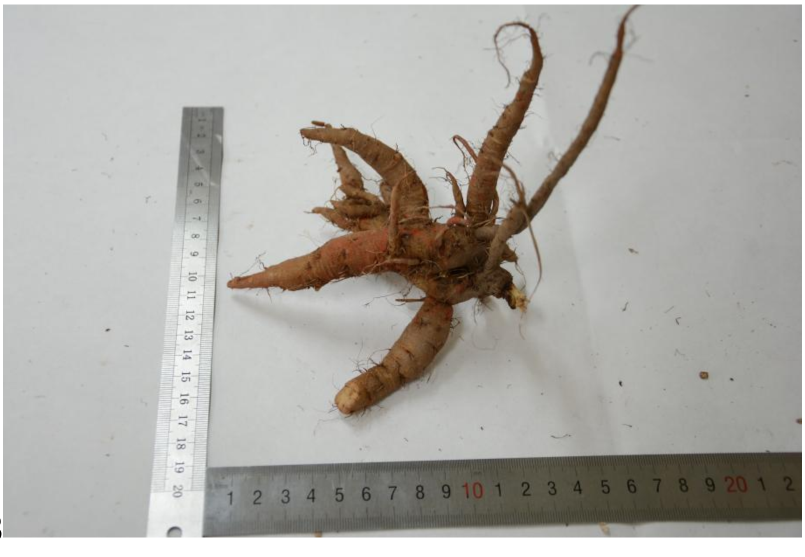
DS13

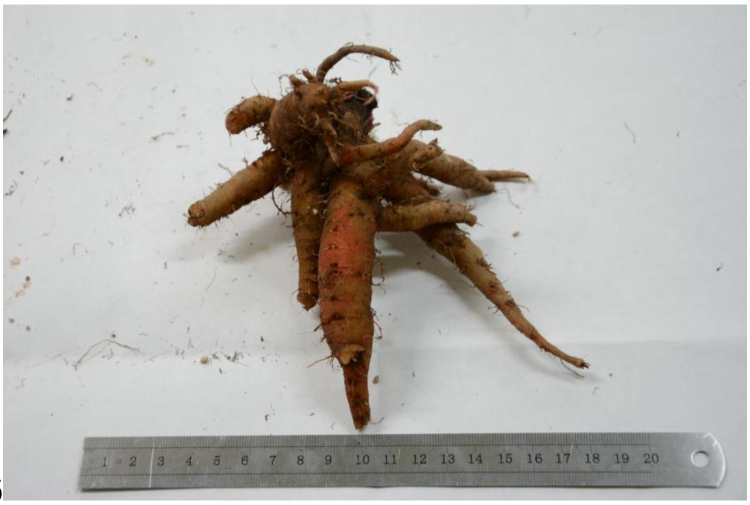

DS16

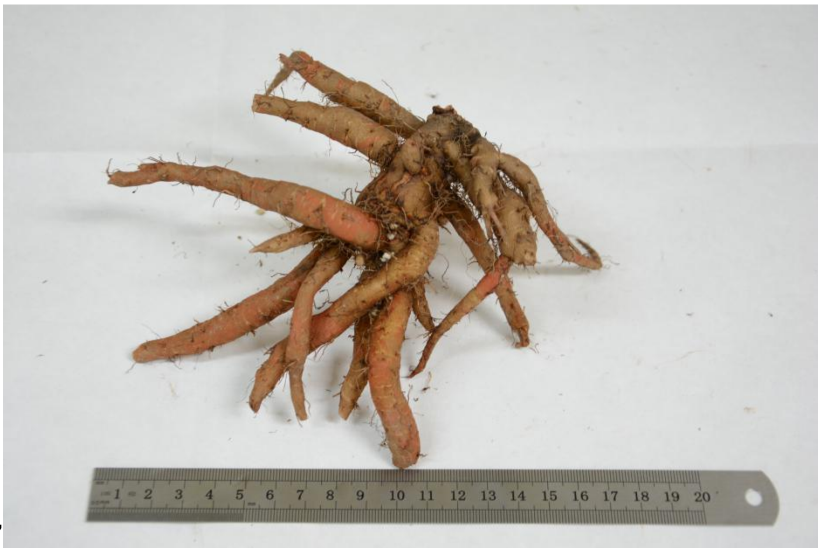

DS17

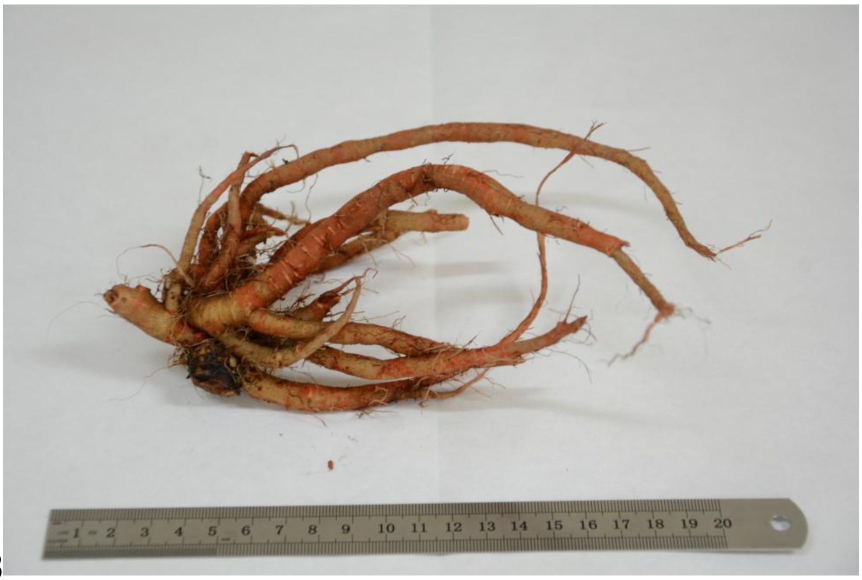

DS18

CK

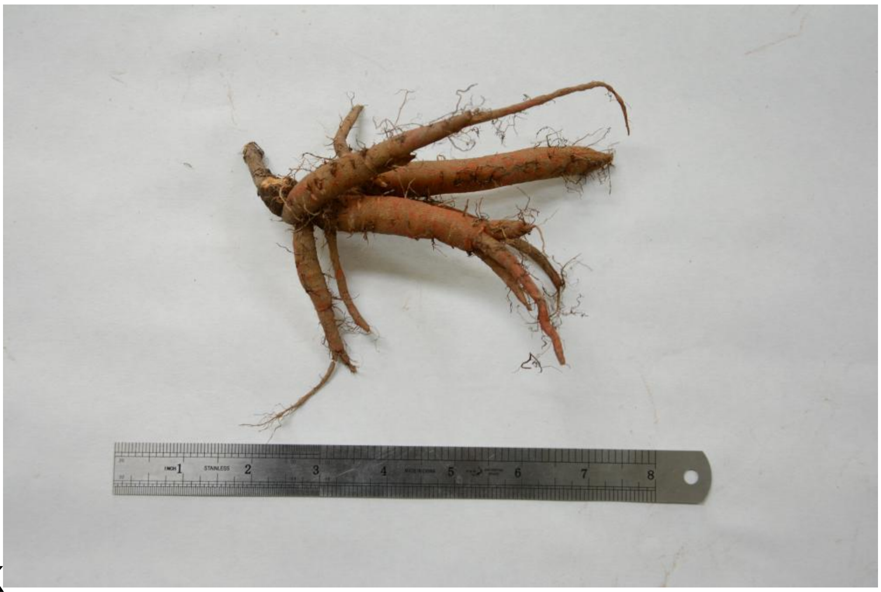

Supplement: Supplemental Information 7 — Photographs of in vitro and greenhouse cultures and photographs of Salvia miltiorrhiza roots in the experiment [file peerj-12-16959-s007.pdf]
